# Sterically Stabilized RIPL Peptide-Conjugated Nanostructured Lipid Carriers: Characterization, Cellular Uptake, Cytotoxicity, and Biodistribution

**DOI:** 10.3390/pharmaceutics10040199

**Published:** 2018-10-23

**Authors:** Chang Hyun Kim, Si Woo Sung, Eun Seok Lee, Tae Hoon Kang, Ho Yub Yoon, Yoon Tae Goo, Ha Ra Cho, Dong Yoon Kim, Myung Joo Kang, Yong Seok Choi, Sangkil Lee, Young Wook Choi

**Affiliations:** 1 College of Pharmacy, Chung-Ang University, 221 Heuksuk-dong, Dongjak-gu, Seoul 156-756, Korea; yj.ch.kim@gmail.com (C.H.K.); woodytow2@naver.com (S.W.S.); esl0409@naver.com (E.S.L.); kth2742@naver.com (T.H.K.); phantomryda@naver.com (H.Y.Y.); rndbsxo5318@naver.com (Y.T.G.); 2 College of Pharmacy, Dankook University, 119 Dandae-ro, Dongnam-gu, Cheonan, Chungnam 330-714, Korea; llpigdreamll@naver.com (H.R.C.); skykimdy@naver.com (D.Y.K.); kangmj@dankook.ac.kr (M.J.K.); analysc@dankook.ac.kr (Y.S.C.); 3 College of Pharmacy, Keimyung University, 1095 Dalgubeol-daero, Daegu 704-701, Korea; skdavid@kmu.ac.kr

**Keywords:** nanostructured lipid carrier, RIPL peptide, cellular uptake, steric stabilization, cytotoxicity, biodistribution

## Abstract

As a platform for hepsin-specific drug delivery, we previously prepared IPLVVPLRRRRRRRRC peptide (RIPL)-conjugated nanostructured lipid carriers (RIPL-NLCs) composed of Labrafil^®^ M 1944 CS (liquid oil) and Precirol^®^ ATO 5 (solid lipid). In this study, to prevent the recognition by the mononuclear phagocyte system, polyethylene glycol (PEG)-modified RIPL-NLCs (PEG-RIPL-NLCs) were prepared using PEG3000 at different grafting ratios (1, 5, and 10 mole %). All prepared NLCs showed a homogeneous dispersion (130–280 nm), with zeta potentials varying from −18 to 10 mV. Docetaxel (DTX) was successfully encapsulated in NLCs: encapsulation efficiency (93–95%); drug-loading capacity (102–109 µg/mg). PEG-RIPL-NLCs with a grafting ratio of 5% PEG or higher showed significantly reduced protein adsorption and macrophage phagocytosis. The uptake of PEG(5%)-RIPL-NLCs by cancer cell lines was somewhat lower than that of RIPL-NLCs because of the PEG-induced steric hindrance; however, the uptake level of PEG-RIPL-NLCs was still greater than that of plain NLCs. In vivo biodistribution was evaluated after tail vein injection of NLCs to normal mice. Compared to RIPL-NLCs, PEG(5%)-RIPL-NLCs showed lower accumulation in the liver, spleen, and lung. In conclusion, we found that PEG(5%)-RIPL-NLCs could be a promising nanocarrier for selective drug targeting with a high payload of poorly water-soluble drugs.

## 1. Introduction

Nanostructured lipid carriers (NLCs) have been introduced as a new generation of lipid-based drug delivery systems, including liposomes and solid lipid nanoparticles [1]. NLCs are composed of solid lipids and liquid oils, which result in a high loading capacity for poorly water-soluble drugs [2]. They possess numerous advantages, such as biocompatibility, controlled drug release, storage stability, and the possibility of large-scale production. However, their clinical applications for effective drug therapy are still very limited because of the lack of selectivity [3]. Recently, to achieve multifunctionality for effective drug targeting, surface modifications of NLCs have been widely tried [4].

In our previous work, we successfully synthesized a 16-mer RIPL peptide (IPLVVPLRRRRRRRRC) as a cell-penetrating and homing peptide [5] and prepared RIPL peptide-conjugated NLCs (RIPL-NLCs) as a platform for hepsin-specific drug delivery [6]. RIPL-NLCs enhanced the intracellular delivery of the anticancer drug docetaxel (DTX) or a fluorescent probe. However, it is well known that most nanoparticles are cleared from circulation by the mononuclear phagocyte system (MPS) because of opsonization and subsequent uptake by the reticuloendothelial system [7,8]. To overcome these limitations, the concept of “stealth NLCs”, designed to reduce the nonspecific protein adsorption and prevent the MPS uptake, has been suggested [9]. Various polymers have been used to increase the stability of NLCs in blood circulation (described as “steric stabilization”) by building a polymeric barrier on the surface of NLCs [10].

Currently, polyethylene glycol (PEG) has been widely used as a polymeric steric stabilizer. PEG’s neutrality, hydrophilicity, molecular flexibility, and non-immunogenicity make it a natural initial choice [11]. For optimizing a PEGylated NLC system, several factors are crucial, such as the type, length, molecular weight, and density of PEG chains [12]. In particular, PEG chains with molecular weights of 2 kDa or higher have been used to achieve a successful surface modification [13,14]. In an earlier study, we stabilized a liposomal nanocarrier by using PEG3000 at 5 mole % [15] since the density of PEG chains has been recognized as an important factor. In particular, at a density between 5 and 9 mole %, polymer chains can effectively block opsonization while maintaining their flexibility [16].

In this study, to reduce the nonspecific protein adsorption and to prevent the recognition by the MPS, PEG-modified RIPL-NLCs (PEG-RIPL-NLCs) were prepared using PEG3000 as a steric stabilizer at different grafting ratios (1, 5, and 10 mole %), with either DTX or a fluorescent probe encapsulated. Physicochemical properties of various NLCs were evaluated, including their particle size, zeta potential (ZP), polydispersity index (PDI), encapsulation efficiency (EE), and drug loading (DL). The conformational structure and drug release properties of NLCs were also characterized. The steric stabilization effect was evaluated by a protein adsorption assay and a macrophage phagocytosis inhibition study. The cellular uptake behavior of PEG-RIPL-NLCs was assessed using hepsin-expressing SKOV3, MCF7, and LNCaP cells, and the uptake mechanism was investigated in the presence of endocytosis inhibitors. Cytotoxicity and hemolytic activity were also assessed. Finally, an in vivo biodistribution study was performed after intravenous injection of the formulations to normal mice.

## 2. Materials and Methods

### 2.1. Materials

DTX (purity > 99%) was kindly provided by Chong Kun Dang Pharm. Co. (Yongin, Korea). Oleoyl macrogol-6 glycerides (Labrafil^®^ M 1944 CS) and glyceryl distearate (Precirol^®^ ATO 5) were received as a gift from Gattefossé (Saint-Priest, France). 1,1′-Dioctadecyl-3,3,3′,3′-tetramethylindocarbocyanine perchlorate (DiI), a micro bicinchoninic acid (BCA) protein assay kit, 5,5′-dithio-bis-(2-nitrobenzoic acid) (DTNB), cysteine hydrochloride anhydrous, phosphate-buffered saline (PBS) tablets, chlorpromazine hydrochloride, sodium azide, amiloride hydrochloride hydrate, and nystatin were purchased from Sigma-Aldrich Chemical Co. (St. Louis, MO, USA). 1,2-Distearoyl-sn-glycero-3-phosphoethanolamine-N-[maleimide(PEG2000)] (DSPE-PEG2000-Mal) and 1,2-distearoyl-sn-glycero-3-phosphoethanolamine-N-[methoxy(PEG3000)] (DSPE-PEG3000) were purchased from Avanti Polar Lipids (Alabaster, AL, USA). The RIPL peptide was synthesized by Peptron Co. (Daejeon, Korea). All other chemicals and reagents purchased from commercial sources were of analytical or cell culture grade.

Cell lines were purchased from the Korean Cell Line Bank (Seoul, Korea). PBS (10×, pH 7.4), and cell culture materials, including Roswell Park Memorial Institute (RPMI) 1640 medium, Dulbecco′s modified Eagle′s medium (DMEM), fetal bovine serum, penicillin-streptomycin, and trypsin-Ethylenediaminetetraacetic acid (EDTA) (0.25%), were obtained from Invitrogen (Carlsbad, CA, USA). Female Institute of Cancer Research (ICR) mice (5-week-old, 20 ± 2 g) were purchased from the Hanlim Experimental Animal Laboratory (Gyeonggi-do, Korea).

### 2.2. Preparations of Various NLC Formulations

**Figure 1:**
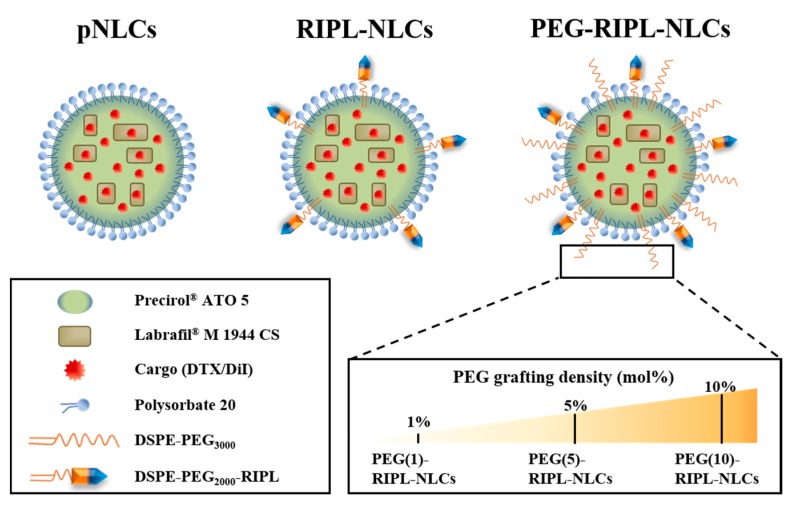
Conformational comparison of different nanostructured lipid carriers (NLCs).

The following DTX-loaded NLC formulations were prepared using a previously published solvent emulsification-evaporation method [6]: plain NLCs (DTX-pNLCs), RIPL-NLCs (DTX-RIPL-NLCs), and PEGylated RIPL-NLCs (DTX-PEG-RIPL-NLCs) (Figure 1). To observe the cellular uptake of NLCs, DiI (a lipophilic red fluorescent probe) was loaded instead of DTX. The unencapsulated DTX or DiI was removed by ultra-centrigufation at 13,000× *g* for 20 min using Amicon^®^ ultra-centrifugal filters (MWCO 100 kDa, Millipore, Billerica, MA, USA). Empty NLCs were prepared without DTX or DiI. All prepared NLCs were stored at 4 °C. The NLC samples were used for the experiments within 2 weeks, and no changes were observed until use.

#### 2.2.1. Preparation of DTX-pNLCs

Labrafil^®^ M 1944 CS (liquid oil), Precirol^®^ ATO 5 (solid lipid), and DTX (or DiI) were dissolved in dichloromethane as an organic phase and then mixed with an aqueous solution containing polysorbate 20 (Tween 20; 1%, *w*/*v*) and polyvinyl alcohol (PVA; 0.5%, *w*/*v*). The mixture was homogenized at 15,000 rpm for 2 min using an Ultra-Turrax^®^ T25 basic homogenizer (IKA Labortechnik, Staufen, Germany), then sonicated using a probe-type sonicator (Sonoplus, HD 2070; Bandelin Electronics, Berlin, Germany) operating at a power of 45% for 3 min under cooling at 5 °C, and finally subjected to solvent evaporation with magnetic stirring at 300 rpm for 3 h.

#### 2.2.2. Preparation of DTX-RIPL-NLCs

The RIPL peptide was conjugated to maleimide-derivatized DTX-NLCs via a thiol-maleimide reaction as reported [6,17]. Briefly, DTX, Precirol^®^ ATO 5, Labrafil^®^ M 1944 CS, and DSPE-PEG2000-Mal (1 mole %) were dissolved in dichloromethane, mixed with the Tween 20/PVA aqueous solution, and then homogenized, sonicated, and evaporated as described above. Subsequently, the RIPL peptide was added to the maleimide-derivatized DTX-NLC dispersion and allowed to react for 12 h at 25 °C. The unreacted RIPL peptide was removed by dialysis against distilled water using a cellulose ester dialysis membrane [50 kDa molecular weight cutoff (MWCO)] for 24 h at 4 °C.

#### 2.2.3. Preparation of DTX-PEG-RIPL-NLCs

PEGylation of RIPL-NLCs was performed by a premodification technique as reported [18]. Briefly, different amounts of DSPE-PEG3000 (1, 5, and 10 mole %) were dissolved in dichloromethane, containing predissolved DTX, Precirol^®^ ATO 5, Labrafil^®^ M 1944 CS, and DSPE-PEG2000-Mal (1 mole %), and homogenized with the Tween 20/PVA aqueous solution, followed by the same procedure as described in Section 2.2.2 for RIPL-NLC preparation. Based on the amount of DSPE-PEG3000 added (1, 5, or 10 mole %), the prepared DTX-RIPL-NLCs were designated PEG(1)-, PEG(5)-, and PEG(10)-RIPL-NLCs, respectively (Figure 1).

### 2.3. Preparation of a Reference DTX Solution

To reproduce a commercial DTX product, Taxotere^®^, a DTX solution (DTX-Sol) was prepared by dissolving DTX at a concentration of 20 mg/mL in distilled water containing polysorbate 80 (25%, *w*/*v*) and ethanol (9.75%, *v*/*v*). DTX-Sol was appropriately diluted with a cell culture medium or normal saline for in vitro cytotoxicity and in vivo biodistribution studies, respectively.

### 2.4. Determination of EE and DL

*EE* and *DL* of NLCs were determined by ultrafiltration using Amicon^®^ ultracentrifugal devices. Briefly, 500 μL of DTX- or DiI-loaded NLC samples was centrifuged for 20 min at 14,000× *g*. The concentration of the free drug in the filtrate was analyzed by high-performance liquid chromatography (HPLC). The following equations were used for the calculations:

*EE* (%) = (*W_T_* − *W_F_*)/*W_T_* × 100 (1)

*DL* (μg/mg) = (*W_T_* − *W_F_*)/*W_L_* (2)

where *W_T_*, *W_F_*, and *W_L_* represent the total amount of the cargo, the amount of free cargo, and the total amount of the lipids, respectively.

### 2.5. HPLC

DTX and DiI were quantified using a Waters Corporation (Milford, MA, USA) HPLC system consisting of a separations module (e2695), an ultraviolet (UV) detector (e2489), and a data station (Empower 3). DTX was separated on a Kromasil^®^ C18 column (5 μm, 4.6 × 250 mm; Akzo Nobel, Bohus, Sweden) using an isocratic mobile phase consisting of acetonitrile and water (55:45, *v*/*v*) at a flow rate of 1 mL/min at 25 °C. The eluate was monitored at a UV wavelength of 230 nm, and the injection volume was 50 μL. For DiI quantification, a fluorescence detector (W2475) was used. Chromatography was performed on a C18 column (5 μm, 4.6 × 150 mm; Shiseido, Tokyo, Japan) at a flow rate of 1.5 mL/min using a mobile phase consisting of 0.05 M dimethyl sulfate and methanol (2:98, *v*/*v*). The injection volume was 50 μL, and the excitation and emission wavelengths were 549 and 565 nm, respectively. The standard calibration curves of DTX and DiI were linear in the ranges of 0.5–100 μg/mL and 0.005–10 μg/mL, respectively, with coefficient of determination (*r*^2^) values of greater than 0.999. The analysis method offered a limit of detection of 0.1 μg/mL for DTX and 0.001 μg/mL for DiI at a signal-to-noise ratio of 3:1.

### 2.6. Particle Size and ZP Analysis

NLC samples were diluted 1:100 in distilled water, which provided an adequate scattering intensity. The particle size, PDI, and ZP of NLCs were measured in triplicate using a dynamic light scattering particle size analyzer (Zetasizer Nano-ZS; Malvern Instruments, Worcestershire, UK).

### 2.7. Transmission Electron Microscopy

NLCs were imaged using a transmission electron microscope (JEM1010; JEOL, Tokyo, Japan) at an acceleration voltage of 80 kV. Briefly, NLC samples were diluted 10-fold with distilled water and placed onto a carbon film grid. The samples were stained with 2% phosphotungstic acid, washed with distilled water, and dried at room temperature before observation.

### 2.8. Conformational Characterization of Ligand Conjugation

The conjugation efficiencies of the RIPL peptide to RIPL-NLCs and PEG-RIPL-NLCs were calculated indirectly by determining the amount of unreacted cysteine in Ellman’s reaction as previously reported [19,20]. Briefly, RIPL-NLCs and PEG-RIPL-NLCs were incubated with a three-fold molar excess of cysteine to block the unreacted maleimide functional groups. Then, DTNB (1 mg/mL) was added to react with the unreacted cysteine, with the formation of a cysteine-5-thio-2-nitrobenzoic acid (TNB) adduct and a concomitant release of an equivalent amount of free TNB. The amount of liberated TNB was analyzed by HPLC at a flow rate of 1.0 mL/min, with UV detection at a wavelength of 420 nm. The mobile phase consisted of a mixture of methanol and a 10 mM ammonium formate solution (5:95, *v*/*v*).

### 2.9. In Vitro Drug Release

An in vitro drug release study was performed using a dialysis bag diffusion method [21]. Briefly, 1 mL of DTX-Sol, DTX-pNLCs, DTX-RIPL-NLCs, or DTX-PEG(5)-RIPL-NLCs was placed in a dialysis bag (100 kDa MWCO; Spectrum Laboratories, Rancho Dominguez, CA, USA). Then, firmly clipped dialysis bags were completely soaked in 90 mL of a release medium [0.5% (*w*/*v*) sodium dodecyl sulfate in PBS, pH 7.4] and incubated at 37 °C with magnetic stirring at 100 rpm. To measure the amount of released DTX, 1 mL of the release medium was withdrawn at predetermined time points (0.25, 0.5, 1, 2, 3, 4, 6, 8, 10, 12, 24, 48, and 72 h), and the dialysate volume was replenished with 1 mL of a fresh release medium. The concentration of DTX in the aliquots was analyzed by HPLC as described above.

### 2.10. Protein Adsorption Assay

Protein adsorption behaviors of the nanocarriers were investigated by measuring the amount of bovine serum albumin (BSA) bound to the NLC surface using a micro BCA protein assay kit as previously reported [22]. Briefly, drug-free NLC formulations were incubated with a PBS solution (pH 7.4) containing BSA (250 μg/mL) at 37 °C for predetermined times (2, 8, 16, and 24 h). After the incubation, samples were centrifuged at 13,000× *g* for 15 min to remove free BSA. The pellets were resuspended in 1 mL of PBS and vortexed, to ensure homogeneity, at 2000 rpm for 20 min. Afterward, 100 µL of the samples was transferred into 96-well plates, mixed with 100 µL of the BCA working reagent, and incubated at 37 °C for 30 min. The absorbance was measured spectrophotometrically at 562 nm using a microplate reader (FlexStation 3; Molecular Devices, Sunnyvale, CA, USA).

### 2.11. Cell Culture

Human ovarian carcinoma (SKOV3), human breast adenocarcinoma (MCF7), and human prostate carcinoma (LNCaP) cells were incubated in RPMI 1640 medium, and murine macrophage (RAW 264.7) cells were incubated in DMEM. Both media contained antibiotics (100 μg/mL streptomycin and 100 U/mL penicillin G) and 10% (*v*/*v*) fetal bovine serum. Cells were cultured every 2–4 days in a humidified incubator in an atmosphere of 5% CO_2_ at 37 °C and 95% relative humidity.

### 2.12. Macrophage Phagocytosis Inhibition Study

Macrophage phagocytosis was analyzed qualitatively and quantitatively using RAW 264.7 cells. Cells were seeded in a Lab-Tek II chamber slide with a cover (Thermo Scientific Nunc, Rochester, NY, USA) at a density of 5 × 10^4^ cells/well for a qualitative assay and a in a 6-well plate at a density of 3 × 10^5^ cells/well for a quantitative assay. After 24 h of incubation, the media were removed; the cells were washed twice with PBS and incubated with culture media containing various DiI-loaded samples (DiI concentration of 100 ng/mL) for 2 h. For qualitative observation, cells were fixed with 4% paraformaldehyde for 15 min, mounted using a mounting medium with 4′,6-diamidino-2-phenylindole (DAPI; Vector Laboratories, Burlingame, CA, USA) to prevent fading and to stain the nuclei, and visualized using a confocal microscope (LSM 700 Meta; Carl Zeiss, Jena, Germany) under a 400× magnification. For quantitative analysis, cells were detached from the wells using trypsin-EDTA, resuspended in 1 mL of PBS, and the mean fluorescence intensity (MFI) was measured using a flow cytometer (FACSCalibur; Becton Dickinson, Franklin Lakes, NJ, USA). Only viable cells were gated and analyzed by counting 10,000 events using the FL2 channel with the CellQuest Pro software (Becton Dickinson, Franklin Lakes, NJ, USA). All experiments were performed in triplicate. Additionally, time-dependent inhibition of phagocytosis was investigated for selected samples [DiI-loaded RIPL-NLCs and PEG(5)-RIPL-NLCs] at predetermined time points (0, 0.5, 2, and 6 h).

### 2.13. Cellular Uptake Study

The intracellular delivery of DiI-loaded NLC formulations was examined qualitatively and quantitatively using the hepsin-expressing SKOV3, MCF7, and LNCaP cancer cell lines incubated in a serum-free RPMI 1640 medium. In addition, the cellular uptake mechanism of the nanocarriers was investigated using endocytosis inhibitors and a low temperature as reported [23]. Briefly, SKOV3 cells were seeded in 6-well plates at a density of 3 × 10^5^ cells/well and preincubated at 37 °C for 1 h in the presence of endocytosis inhibitors such as sodium azide (1 mg/mL), amiloride (500 μg/mL), chlorpromazine (10 μg/mL), and nystatin (25 μg/mL), as well as at a lower temperature (4 °C). Untreated cells served as controls. Following preincubation, the cells were treated with DiI-loaded RIPL-NLCs and PEG(5)-RIPL-NLCs, and MFI was determined as described above.

### 2.14. Cytotoxicity Assessment

In vitro cytotoxicity of DTX-free and DTX-loaded formulations was determined by the (Water-soluble tetrazolium salt) WST-1 assay, as previously reported [24]. Briefly, SKOV3, MCF7, and LNCaP cells were seeded in RPMI 1640 medium into 96-well plates at a density of 5000 cells per well. After reaching 70–80% confluence, the cells were incubated with the growth medium containing DTX-free or DTX-loaded NLC formulations (DTX-equivalent concentrations of 0.1, 1, 10, 100, 500, and 1000 ng/mL) at 37 °C for 24 h. Then, the cells were washed with PBS and incubated with a 10% WST-1 reagent (EZ-cytox; Daeil Lab Service, Seoul, Korea) for 30 min at 37 °C. The absorbance of the WST-1 formazan dye was measured at 450 nm using a microplate reader. Cell viability was measured as a percentage of viable cells relative to the untreated control. Half-maximal inhibitory concentration (IC_50_) values were determined by plotting cell viability against the DTX-equivalent concentration on a log scale.

### 2.15. Hemolysis Test

The hemolytic activity of NLCs was determined as described previously to evaluate their biocompatibility for potential applications via intravenous injection [25]. Fresh blood was collected from ICR mice into heparin-treated tubes and was immediately centrifuged at 1000× *g* for 15 min at 4 °C to separate red blood cells (RBCs). RBCs were washed twice with an isotonic saline solution at a 1:4 (*v*/*v*) ratio by centrifugation at 1000× *g* for 15 min and afterward resuspended, diluted with the saline solution, and stored at 4 °C. The obtained RBC suspension (2%) was used for the assay within 24 h.

DTX-free and DTX-loaded formulations were diluted with the saline solution to a DTX-equivalent concentration of 10 μg/mL and mixed with the RBC suspension at a 1:1 (*v/v*) ratio. The saline solution with/without Triton X-100 (1%, *v/v*) was used as a positive control (100% hemolysis) and a negative control (0% hemolysis), respectively. Samples were incubated in a shaking incubator (SI-900R; Jeio Tech, Gyeonggi-do, Korea) at 37 °C for 1 h at 100 rpm, followed by centrifugation at 1500× *g* for 10 min, and the tubes were then photographed. To quantify the amount of hemoglobin released, absorbance of the supernatant was measured spectrophotometrically at 540 nm using a microplate reader. The rate of hemolysis (%) was calculated as (*A* − *A*_0_)/(*A*_100_ − *A*_0_) × 100, where *A* is the absorbance of the sample, while *A*_0_ and *A*_100_ are the absorbance values of the negative and positive control, respectively.

### 2.16. Biodistribution

#### 2.16.1. Administration of Formulations and Tissue Sampling

The animal experiment was approved by the Institutional Animal Care and Use Committee of Chung-Ang University (2018-00090, 3 August 2018, Seoul, Korea) and was carried out in accordance with the National Institute of Health Guidelines for the Care and Use of Laboratory Animals. Female ICR mice were randomly divided into three groups (*n* = 15–20) and injected intravenously via the tail vein with DTX-Sol, DTX-RIPL-NLCs, or DTX-PEG-RIPL-NLCs at a dose of 5 mg DTX-equivalent/kg. At 0.5, 2, and 6 h after the injection, the mice were anesthetized, and 0.5 mL of blood was collected from the retro-orbital plexus into a heparinized tube. The blood samples were centrifuged at 13,000× *g* for 20 min to separate the plasma, which was frozen at −80 °C until analysis. After blood collection, the mice were sacrificed, and the lung, heart, liver, spleen, and kidneys were harvested, washed with PBS, weighed after removing excess fluid, and preserved in a −80 °C freezer.

#### 2.16.2. Sample Pretreatment

The frozen tissues were thawed at room temperature and sectioned. The sections were weighed and mixed with PBS at a ratio of 1:2 (*w*/*v*) for the liver, lung, and kidney and 1:4 (*w*/*v*) for the heart and spleen. The mixtures were placed into 2 mL tubes with four steel beads and homogenized at 3000× *g* using a Bead Blaster 24 homogenizer (Benchmark Scientific, Edison, NJ, USA) twice for 60 s each with a 15 s interval. The resulting homogenates were stored at −80 °C until analysis. For analysis of DTX, 100 μL of tissue homogenates or plasma samples were mixed with 20 μL of methanol and 900 μL of ether. The samples were vortexed for 5 min and centrifuged at 13,000 rpm for 5 min at 4 °C. The organic phase was transferred to another tube and evaporated to dryness at 40 °C under a stream of N_2_ gas. The residue was reconstituted in 100 μL of 30% acetonitrile, and the samples were analyzed by liquid chromatography-tandem mass spectrometry (LC-MS/MS).

#### 2.16.3. DTX Determination by LC-MS/MS

An atmospheric pressure ionization (API) 2000 triple quadrupole mass spectrometer (ABI SCIEX, Foster City, CA, USA) and an LC-20 Prominence HPLC system (Shimadzu, Tokyo, Japan) were used for LC-MS/MS analysis of DTX. The systems were interfaced through electrospray ionization (ESI) in a positive ion mode. For LC, a Phenomenex Luna C18 column (2.0 × 150 mm, 5 μm) and an isocratic mobile phase consisting of 15% water and 85% acetonitrile (*v*/*v*) were used. The autosampler and the column oven were maintained at 4 and 40 °C, respectively. The ESI source parameters were set as follows: spray voltage, 5500 V; spray temperature, 350 °C; gas 1, 30 psi; gas 2, 34 psi; collision gas, 6 psi; curtain gas, 16 psi. For sensitive and selective identification of DTX, multiple reaction monitoring (MRM) was carried out. As the precursor ion for MRM, the Na adduct ion of DTX, observed at 829.9 *m*/*z*, was selected. The MRM precursor/product ion transitions used for DTX in the present study were *m*/*z* 829.9/548.9 (screening transition), 829.9/303.8 (the first confirmatory transition), and 829.9/248.0 (the second confirmatory transition). All data were acquired and analyzed using Analyst, version 1.5.2 (ABI SCIEX, Foster City, CA, USA).

### 2.17. Statistical Analysis

All values were expressed as the mean ± standard deviation (SD) (*n* = 3). Data were analyzed through statistical product and service solutions (SPSS) 19.0 software (USA). Statistical significance was determined using the analysis of variance (ANOVA) followed by a Tukey’s test for post hoc multiple comparisons, and differences were considered significant at *p* < 0.05.

## 3. Results

### 3.1. Characterization of NLCs

Physicochemical characteristics of various NLCs were evaluated, including their particle size, PDI, ZP, EE, and DL (Table 1). The average sizes of NLCs ranged from approximately 130 to 280 nm, as determined by dynamic light scattering. There were no significant changes in the particle size after encapsulation of DTX or DiI. Compared with that of pNLCs, the particle sizes of RIPL-NLCs and PEG-RIPL-NLCs were significantly increased by the modification with the RIPL peptide or PEG, owing to the increased hydrodynamic diameter [26]. However, as the grafting density of PEG increased, the particle size decreased. All formulations had PDI values below 0.4, indicating a homogeneous particle size distribution. Regarding ZP, all pNLCs were negatively charged in the range of −14.1 mV to −17.7 mV, whereas all RIPL-NLCs showed positive charges, ranging from 6.8 to 9.9 mV. The ZP changes could be attributed to the positive charge of the RIPL peptide, which contains arginine residues in the sequence. Compared to RIPL-NLCs, PEG-RIPL-NLCs showed a decreasing tendency in the surface charge, depending on the degree of PEGylation. All NLCs showed high EE and DL values for either DTX or DII—91.8–94.5% and 102.3–109.1 μg/mg for DTX; 99.5–99.7% and 1.9–2.1 μg/mg for DiI, respectively—revealing negligible differences between pNLCs and surface-modified NLC formulations.

**Figure 2:**
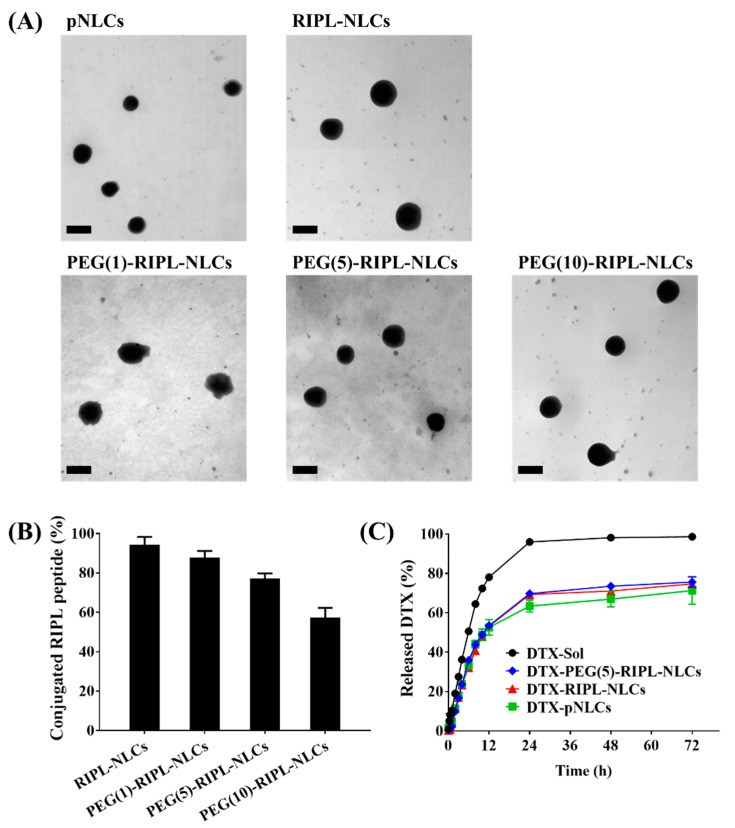
Characterization of various NLCs. (**A**) TEM images of docetaxel (DTX)-loaded NLCs. Scale bar = 200 nm. (**B**) Conjugation efficiencies of the RIPL peptide. Data represent the mean ± SD (*n* = 3). (**C**) In vitro release profiles of DTX from various formulations. Data are the mean ± SD (*n* = 3).

Transmission electron microscopy (TEM) revealed that particles in the NLC formulations were spherical and had a diameter of approximately 150–200 nm, without any aggregation, which implied their high colloidal stability (Figure 2A). These sizes were somewhat smaller than the hydrodynamic diameters determined by dynamic light scattering, probably due to the dehydration during sample preparation for microscopic observation [27]. The degree of RIPL peptide conjugation on the surface of various NLCs is presented in Figure 2B. RIPL-NLCs showed the highest value (94.4 ± 4.0%), while PEGylation decreased the conjugation efficiency. As the amount of PEG chains increased, the degree of conjugation decreased, resulting in the order of PEG(1)-RIPL-NLCs (87.9 ± 3.3%) > PEG(5)-RIPL-NLCs (77.3 ± 2.5%) > PEG(10)-RIPL-NLCs (57.4 ± 4.9%). The in vitro DTX release from different DTX-loaded formulations is shown in Figure 2C. The drug release from DTX-Sol reached approximately 80% within 12 h, whereas that from pNLCs, RIPL-NLCs, and PEG(5)-RIPL-NLCs was 51, 53, and 53%, respectively, for the same period of time. Compared to the fast release from DTX-Sol, the drug release from all NLC formulations was slow and sustained, with a fast release in the initial 12 h, followed by a sustained release afterward.

### 3.2. Protein Adsorption

**Figure 3:**
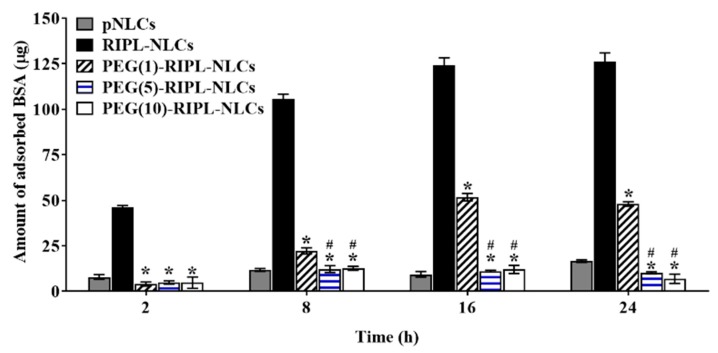
Bovine serum albumin (BSA) adsorption on various NLCs as a function of time. * *p* < 0.05 versus RIPL peptide-conjugated NLCs (RIPL-NLCs); ^#^ *p* < 0.05 versus PEG (1 mole %)-grafted RIPL-NLCs (PEG(1)-RIPL-NLCs).

The potential plasma protein anti-adsorption of NLC formulations was evaluated using BSA, the most abundant protein in serum, as a model plasma protein [28]. As shown in Figure 3, the protein adsorption was time-dependent and varied among NLCs. The amount of protein adsorbed on pNLCs after 2 h incubation was 7.9 ± 1.3 μg (approximately 3% of the initially added BSA), which increased to 16.6 ± 0.7 μg by 24 h. In contrast, RIPL-NLCs strongly interacted with the protein, showing values of 46.3 ± 0.9 μg at 2 h and 126.3 ± 4.8 μg at 24 h. Compared to RIPL-NLCs, PEG-RIPL-NLCs showed reduced adsorption, although the degree of adsorption varied with the grafting ratio of PEG molecules. As the PEG ratio increased above 5 mole %, the protein adsorption was significantly reduced at all time points and constituted 4.9 ± 0.9 μg and 4.8 ± 3.2 μg at 2 h and 10.2 ± 0.6 μg and 6.9 ± 2.6 μg at 24 h for PEG(5)-RIPL-NLCs and PEG(10)-RIPL-NLCs, respectively. Meanwhile, PEG(1)-RIPL-NLCs showed a different pattern. The adsorption level for PEG(1)-RIPL-NLCs at 2 h (4.1 ± 1.3 μg) was similar to those of PEG(5)-RIPL-NLCs and PEG(10)-RIPL-NLCs but increased to 22.3 ± 1.7 μg at 8 h, 51.7 ± 2.0 μg at 16 h, and 48.1 ± 1.2 μg at 24 h. Consequently, PEGylation at 1% was not effective in blocking protein adsorption for a long period but could only provide temporary protection. Thus, for a sufficient anti-adsorption effect, modification with more than 5 mole % PEG was necessary.

### 3.3. Inhibition of Phagocytosis

**Figure 4:**
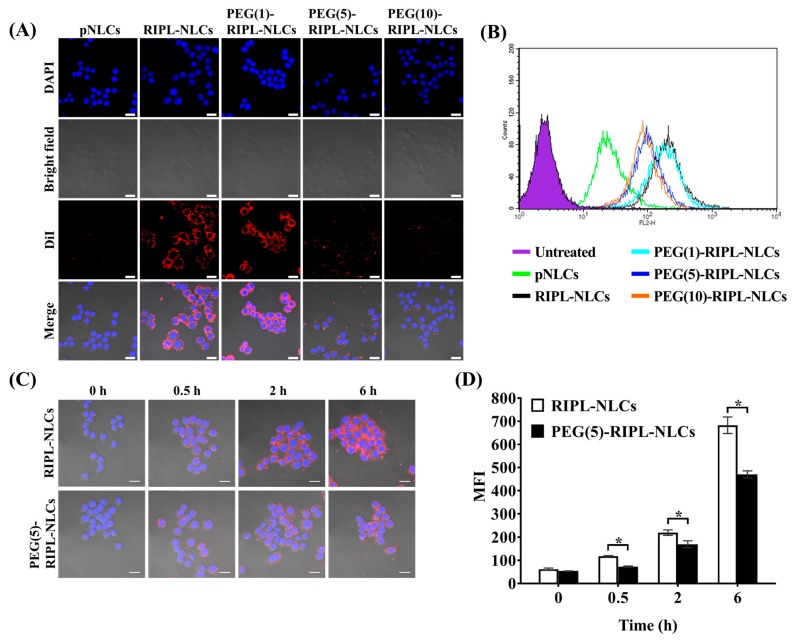
Phagocytic uptake of various 1,1′-dioctadecyl-3,3,3′,3′-tetramethylindocarbocyanine perchlorate (DiI)-loaded NLCs by RAW 264.7 macrophage cells. (**A**) Confocal laser scanning microscopy (CLSM) images after 2 h incubation. The nucleus is stained with 4′,6-diamidino-2-phenylindole (DAPI) (blue), and DiI is distributed in the cells (red). Scale bar = 20 μm. (**B**) Flow cytometry histogram of phagocytosed DiI-loaded NLCs after 2 h incubation. (**C**) CLSM images acquired after treatment with RIPL-NLCs and PEG(5)-RIPL-NLCs for different times. Scale bar = 20 μm. (**D**) MFI values calculated after treatment with RIPL-NLCs and PEG (5 mole %)-grafted RIPL-NLCs (PEG(5)-RIPL-NLCs). Data are the mean ± SD (*n* = 3). * *p* < 0.05.

Phagocytosis of various NLCs by murine macrophages (RAW 264.7) was studied using DiI as a fluorescent probe. Confocal microscopy (Figure 4A) showed that the uptake of pNLCs by macrophages was negligible, whereas that of RIPL-NLCs was obvious, based on the bright and intense fluorescence. However, the macrophage phagocytosis was markedly reduced by PEGylation, especially at a PEG density of more than 5 mole %. These results were consistent with the above protein binding data. Quantitative analysis of the NLC uptake by macrophages using flow cytometry (Figure 4B) showed that the MFI values for NLC formulations were in the order of RIPL-NLCs ≥ PEG(1)-RIPL-NLCs > PEG(5)-RIPL-NLCs ≥ PEG(10)-RIPL-NLCs > pNLCs. Although the surface modification of NLCs with the RIPL peptide increased their uptake by macrophages, PEG at more than 5 mole % effectively inhibited phagocytosis. Thus, to compare the steric stabilization effects, PEG(5)-RIPL-NLCs were selected as a platform system for further evaluation.

Time dependency of the uptake of NLCs by macrophages was investigated by incubation of RAW 264.7 cells with DiI-loaded RIPL-NLCs and PEG(5)-RIPL-NLCs for different times (Figure 4C). As expected, no uptake was observed at 0 h. At 0.5 h, low and intermittent fluorescence was observed for both NLCs. However, after 2 h, the fluorescence intensity greatly increased for both NLCs. In particular, at 6 h, RIPL-NLCs showed greater phagocytosis compared with that of PEG(5)-RIPL-NLCs. Furthermore, MFI values of both NLC formulations were compared for quantitative evaluation (Figure 4D). At all the time points, a significant reduction in phagocytosis of PEG(5)-RIPL-NLCs by RAW 264.7 cells was observed. Notably, at 6 h, the uptake of PEG(5)-RIPL-NLCs by macrophages was 1.4-fold less than that of RIPL-NLCs.

### 3.4. In Vitro Cellular Uptake

**Figure 5:**
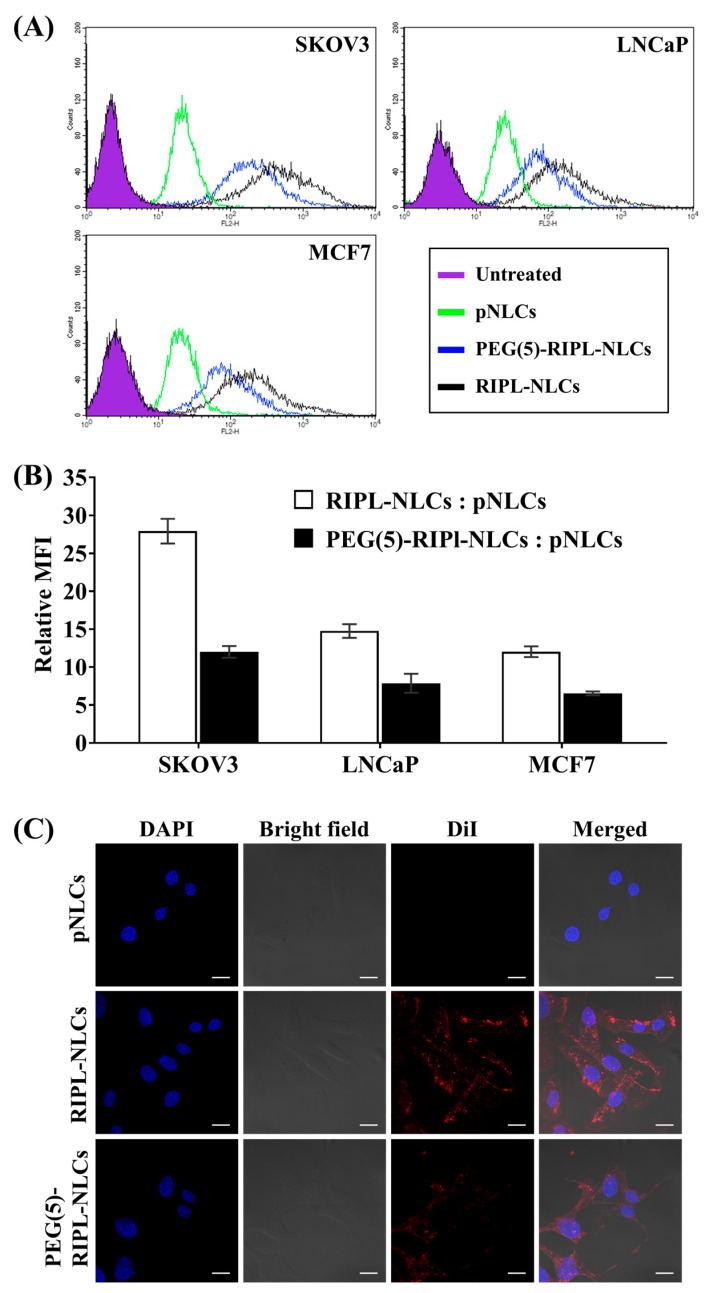
Cellular uptake of DiI-loaded NLCs by the SKOV3, MCF7, and LNCaP cell lines. (**A**) Flow cytometry histogram of plain NLCs (pNLCs), RIPL-NLCs, and PEG(5)-RIPL-NLCs uptake. (**B**) The relative MFI values of treatments: RIPL-NLCs versus pNLCs and PEG(5)-RIPL-NLCs versus pNLCs. Data are the mean ± SD (*n* = 3). (**C**) CLSM images of SKOV3 cells incubated with DiI-loaded NLCs. Scale bar = 20 μm.

Intracellular delivery of DiI-loaded NLCs was evaluated using the SKOV3, LNCaP, and MCF7 cell lines. As shown in a flow cytometry histogram (Figure 5A), the degree of the fluorescence peak shift was in the order of RIPL-NLCs > PEG(5)-RIPL-NLCs > pNLCs in all cell lines. For further quantitative comparison, relative MFI values were calculated for surface-modified NLCs and pNLCs (Figure 5B). In SKOV3 cells, the relative MFI values showed 27- and 12-fold increases for RIPL-NLCs and PEG(5)-RIPL-NLCs, respectively, indicating greater selectivity of SKOV3 cells than that of the other cell lines. In the LNCaP and MCF7 cell lines, the increases were approximately 13- and 7-fold for RIPL-NLCs and PEG(5)-RIPL-NLCs, respectively. Internalization of DiI-loaded NLCs by SKOV3 cells was further visualized by confocal microscopy (Figure 5C). Merged DAPI-stained and bright-field images showed that RIPL-NLCs were greatly internalized into SKOV3 cells, owing to the cell-penetrating effect of the RIPL peptide. Although the fluorescence intensity of PEG(5)-RIPL-NLCs was weaker than that of RIPL-NLCs, it was still stronger than that of pNLCs.

### 3.5. Cellular Uptake Mechanism

**Figure 6:**
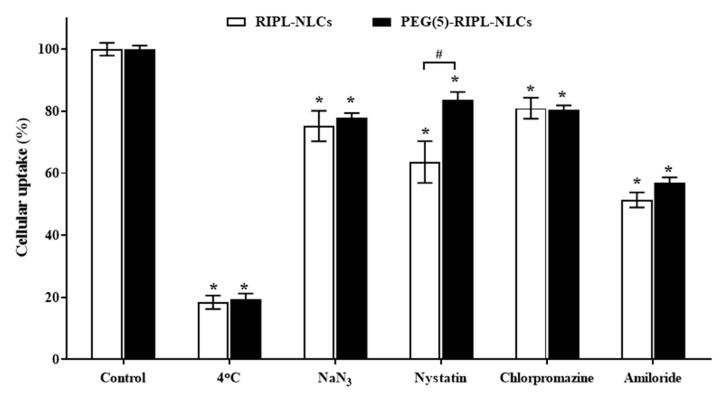
Competitive inhibition study of cellular uptake of RIPL-NLCs and PEG(5)-RIPL-NLCs by SKOV3 cells in the presence of endocytosis inhibitors and at a low temperature. Data are the mean ± SD (*n* = 3). * *p* < 0.05 versus control; ^#^ *p* < 0.05 between RIPL-NLCs and PEG(5)-RIPL-NLCs.

To determine the mechanism of RIPL-NLC and PEG(5)-RIPL-NLC uptake, SKOV3 cells were preincubated with a number of endocytosis inhibitors and were challenged with a low temperature. Compared with that in the negative control (without pretreatment), the cellular uptake was reduced in all other cases, even though the levels of inhibition were different (Figure 6). At a low temperature (4 °C), the uptake of both NLC formulations was greatly reduced (approximately 80% inhibition), indicating the energy-dependent endocytosis. This phenomenon was further elucidated by pretreatment with sodium azide, which resulted in the reduction of uptake levels to approximately 80% of control for both NLC formulations. Pretreatment with nystatin, a blocking agent for caveolin-mediated endocytosis, reduced the uptake of RIPL-NLCs and PEG(5)-RIPL-NLCs to 63% and 83%, respectively, showing a significant difference (*p* = 0.016) between the formulations. Pretreatments with chlorpromazine (clathrin-mediated endocytosis inhibitor) and amiloride (macropinocytosis inhibitor) decreased the uptake of both NLC samples to approximately 80% and 51–57%, respectively, revealing no significant difference between the two NLC formulations.

### 3.6. Cytotoxicity

**Figure 7:**
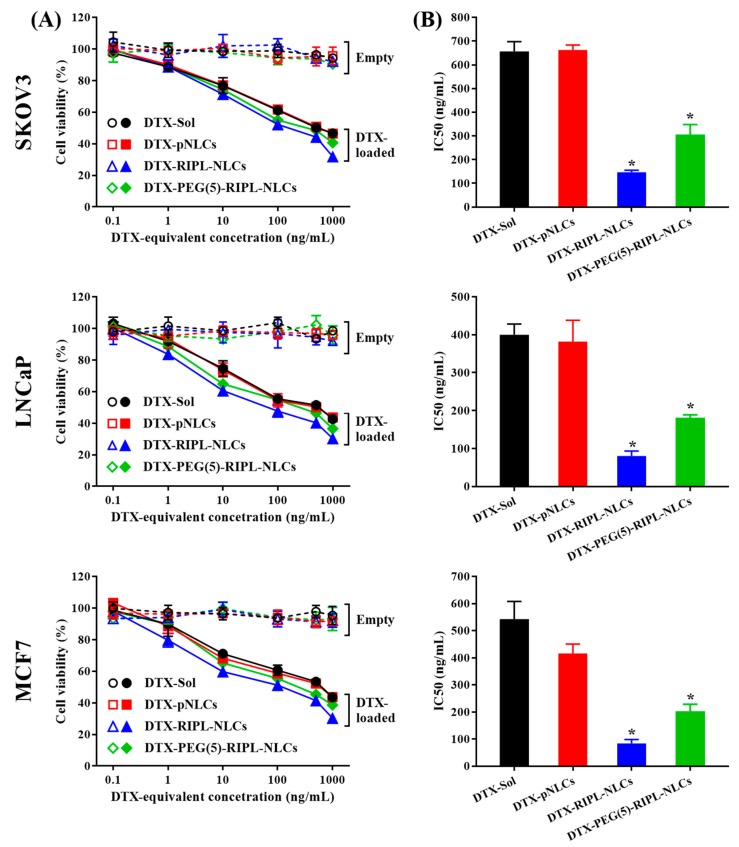
Cytotoxicity of the empty and DTX-loaded formulations for SKOV3, MCF7, and LNCaP cells. (**A**) Cell viability plotted against the log DTX-equivalent concentration. DTX-Sol (○,●); DTX-pNLCs (□,∎); DTX-RIPL-NLCs (△,▲); DTX-PEG(5)-RIPL-NLCs (◊,♦). Opened for empty formulations and filled for DTX-loaded formulations; (**B**) IC_50_ values of DTX-loaded formulations. Data are the mean ± SD (*n* = 3). * *p* < 0.05 versus DTX-pNLCs.

Cytotoxicity of the empty and DTX-loaded NLCs was evaluated for SKOV3, LNCaP, and MCF7 cells after 24 h incubation. As shown in Figure 7A, all empty NLC formulations (dashed lines) showed relatively low cytotoxicity at all concentrations, with the cell viability of more than 95%, regardless of the cell type. On the other hand, the DTX-loaded NLC formulations (solid lines) exhibited cytotoxic effects in a dose-dependent manner. To further compare the formulation effects, IC_50_ values for different DTX-loaded NLCs were calculated (Figure 7B). In the case of DTX-pNLCs, the IC_50_ values were 667.6 ± 112.5 ng/mL, 400.9 ± 130.3 ng/mL, and 432.2 ± 109.9 ng/mL for SKOV3, LNCaP, and MCF7 cells, respectively, and were not significantly different from those of DTX-Sol. In contrast, the IC_50_ values of DTX-RIPL-NLCs and DTX-PEG(5)-RIPL-NLCs were markedly lower for all the cell lines and significantly different from those of DTX-pNLCs. In particular, DTX-RIPL-NLCs showed the lowest IC_50_ values, ranging from 80.2 to 146.2 ng/mL, for all the cell lines, and these values were significantly lower than those of DTX-PEG(5)-RIPL-NLCs. Although PEGylation weakened the cytotoxic effects of DTX-RIPL-NLCs, the IC_50_ values of DTX-PEG(5)-RIPL-NLCs for the cell lines tested still remained in the range from 181.3 to 305.8 ng/mL, i.e., were more than twice as low as those of DTX-pNLCs.

### 3.7. Hemocompatibility

**Figure 8:**
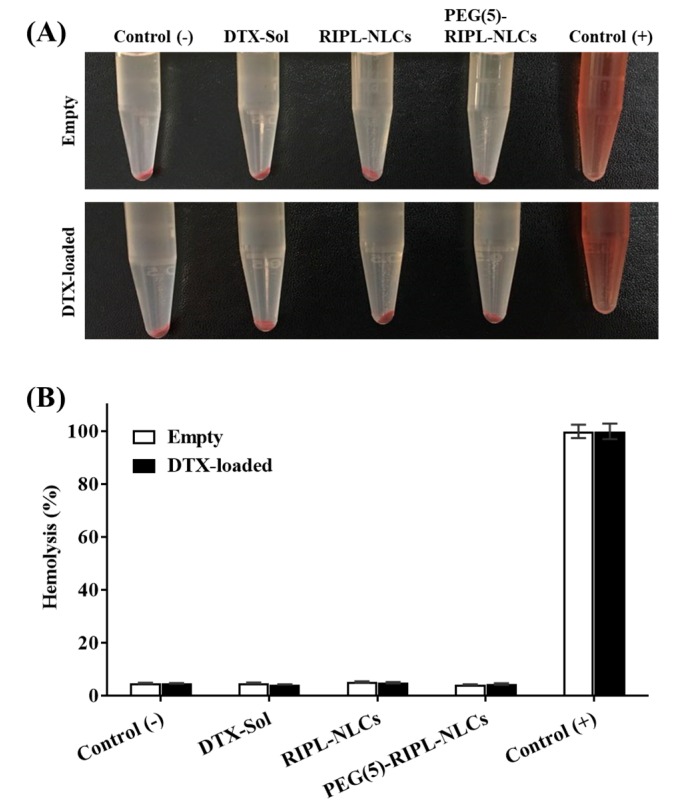
Hemocompatibility of the empty and DTX-loaded formulations with red blood cells (RBCs). (**A**) Photographs of tubes after centrifugation. (**B**) Percentage of hemolysis within 1 h. A saline solution was used as the negative control, and Triton X-100 (1%, *v*/*v*) was added to the positive control. Data are the mean ± SD (*n* = 3).

Hemocompatibility of the empty and DTX-loaded NLC formulations was evaluated by incubating the samples with an RBC suspension. As shown in Figure 8, complete hemolysis of RBCs was observed in the positive control (Triton X-100-treated), whereas hemolysis was negligible (<5%) in the negative control (saline-treated). All the tested formulations, including DTX-Sol, RIPL-NLCs, and PEG(5)-RIPL-NLCs, showed negligible hemolysis, similar to that in the negative control, indicating biocompatibility of the NLC formulations.

### 3.8. In Vivo Biodistribution

**Figure 9:**
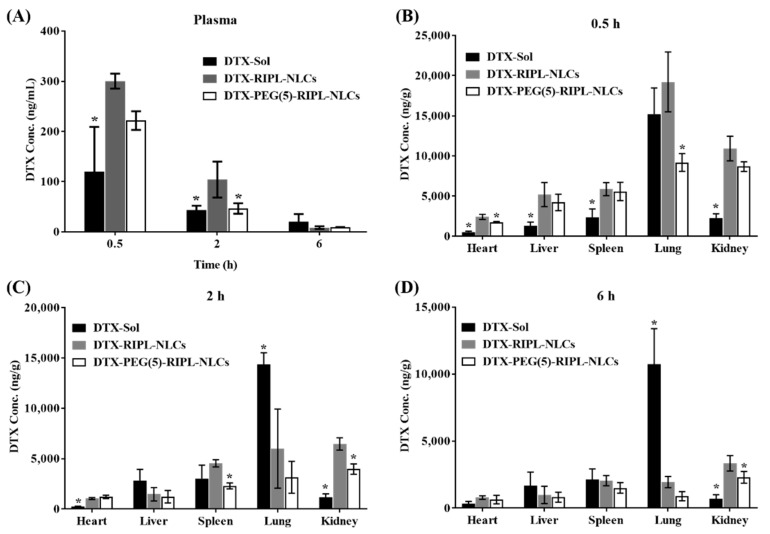
Biodistribution of DTX-loaded formulations in Institute of Cancer Research (ICR) mice after tail vein injection at a dose of 5 mg DTX-equivalent/kg (∎: DTX-Sol; ∎: DTX-RIPL-NLCs; □: DTX-PEG(5)-RIPL-NLCs). Concentrations of DTX in the plasma (**A**) and representative organs are presented at different time points (0.5 h (**B**), 2 h (**C**), and 6 h (**D**)). Data are the mean ± SD (*n* = 5). * *p* < 0.05 versus DTX-RIPL-NLCs.

In vivo tissue distribution of DTX-Sol, DTX-RIPL-NLCs, and DTX-PEG(5)-RIPL-NLCs was investigated after tail vein injection of the formulations to normal mice (Figure 9). In the plasma and all harvested organs, the highest DTX levels were detected at 0.5 h after administration, and the levels then gradually decreased over time. At 0.5 h, the DTX-RIPL-NLC- and DTX-PEG(5)-RIPL-NLC-injected mice exhibited higher plasma DTX concentrations than did the DTX-Sol-injected animals. However, at 2 h, the plasma DTX concentration was much higher in the DTX-RIPL-NLC-treated mice than in either DTX-Sol-or DTX-PEG(5)-RIPL-NLC-treated animals. Following injection, DTX disappeared from the blood circulation within 6 h. Meanwhile, the study of biodistribution into major organs showed that DTX-Sol was predominantly distributed to the lung, and this pattern was maintained for 6 h. DTX-RIPL-NLCs accumulated at relatively high levels in the lung and kidney. In comparison, DTX-PEG(5)-RIPL-NLCs were distributed evenly in the lung, heart, kidney, and liver. Regarding the 2 h clearance of DTX-RIPL-NLCs and DTX-PEG(5)-RIPL-NLCs, the DTX concentrations drastically decreased in the lung, kidney, and liver. In the spleen, the DTX concentration did not change for DTX-RIPL-NLCs but decreased for DTX-PEG(5)-RIPL-NLCs. Overall, the biodistribution patterns of the two NLC formulations were not significantly different, even though appreciable fluctuations were observed.

## 4. Discussion

In the present study, RIPL-NLCs were sterically stabilized with PEG3000 (PEG-RIPL-NLCs) to prevent the recognition by MPS. The particle sizes of RIPL-NLCs (260 to 277 nm) were reduced by PEGylation (178 to 238 nm). As the modification ratio of PEG3000 increased from 1 to 10 mole %, the sizes of PEG-RIPL-NLCs decreased, which could be due to the amphiphilic nature of DSPE-PEG3000, leading to the reduction of the interfacial tension between the aqueous and organic phase during preparation [29]. While nanoparticles with hydrodynamic radii of <10 nm can be rapidly cleared from the systemic circulation by renal filtration, NLC formulations of approximately 200 nm may effectively accumulate in tumors via the enhanced permeability and retention effect [30]. In comparison to RIPL-NLCs, PEG-RIPL-NLCs showed a decreased tendency for surface charges, depending on the degree of PEGylation, probably due to reduced surface exposure of RIPL peptide molecules, which were overlaid with longer PEG segments [31]. In general, accumulation of PEG3000 on the surface of NLCs may attract water molecules to the interior of the lipid-based carrier, resulting in the modulation of drug release [32]. In this study, the surface modification of NLCs with the RIPL peptide and/or PEG3000 somewhat increased the DTX release. However, there was no significant difference between them, possibly due to the high solubilizing capacity of the release medium containing sodium dodecyl sulfate, which increased the wetting of nanoparticle, thereby facilitating the DTX dissolution.

We investigated steric stabilization of NLCs, depending on the PEG grafting density, by evaluating the protein adsorption by NLCs and the uptake of NLCs by macrophages. The matrix-type carrier, pNLCs, exhibited low protein adsorption and a negligible uptake by macrophages. This serum stability and phagocytosis avoidance of pNLCs are possibly due to the hydrophilic surfactant (polysorbate 20), which stabilizes the surface [33]. However, in the case of RIPL-NLCs, large protein amounts were rapidly adsorbed, resulting in high phagocytosis. This high protein adsorption seems to be mainly due to the van der Waals interaction between the positively charged RIPL peptide, exposed on the NLC surface, and BSA [15]. PEG-RIPL-NLCs showed increases in the inhibition of protein adsorption and phagocytosis as the PEG density increased. This stealth effect of PEGylation may be explained by steric repulsion and high mobility of longer PEG chains, which may prevent the RIPL peptide from interacting with serum proteins [11]. In general, 1.5–5-kDa PEG chains, with a grafting density of 5–10 mole %, show a good stealth effect with increased hydrophilicity [8,34]. Furthermore, PEG chains can acquire different conformations, depending on the grafting density [35]. At a low grafting density (up to 4 mole %), PEG chains show a “mushroom” conformation, whereas a “brush” conformation is acquired at a higher grafting density [36]. The ideal coverage to provide the most effective opsonin repulsion has been described as intermediate between these configurations [37]. Considering factors such as the molecular weight, grafting density, and the conformation of PEG, the modification with 5 mole % PEG3000 was selected to achieve an efficient surface coverage of RIPL-NLCs.

The in vitro cellular uptake study provided some evidence of the advantages of RIPL peptide-modified NLC formulations for effective internalization into cancer cells. After PEGylation, the degree of internalization of PEG(5)-RIPL-NLCs was reduced, but the uptake pathway was not greatly changed. The cellular uptake efficiencies of RIPL-NLCs and PEG(5)-RIPL-NLCs were remarkably higher than that of pNLCs, demonstrating that the RIPL peptide facilitated the uptake by hepsin-expressing target cells, which could be attributed to the cell-penetrating and homing function of the RIPL peptide, as previously reported [5]. However, because of the shielding effect of PEG, which interferes with the ligand–receptor interaction [38], the cellular uptake of PEG(5)-RIPL-NLCs was lower than that of RIPL-NLCs. On the other hand, to investigate the uptake mechanism of RIPL-modified NLC formulations, several inhibitors, such as nystatin, chlorpromazine, sodium azide, and amiloride, as well as a low temperature, were tested. It is well-known that the endocytic uptake is an energy-dependent process [39]. The results of competition between low temperature and a metabolic inhibitor (sodium azide) displayed a strong impact on the cellular uptake, indicating that the uptake process of both NLC formulations was energy-dependent. Moreover, internalization was significantly restrained by pretreatment with amiloride, nystatin, and chlorpromazine. Among these inhibitors, amiloride caused the greatest inhibition, indicating that macropinocytosis may be the main mechanism of endocytosis of NLCs. Additionally, both caveolin- and clathrin-mediated endocytosis pathways are involved because pretreatments with nystatin and chlorpromazine showed inhibitory effects. In particular, nystatin pretreatment greatly inhibited the uptake of RIPL-NLCs, showing a significant difference from that of PEG(5)-RIPL-NLCs. Thus, we could conclude that PEGylation affected the caveolin-mediated uptake of RIPL-NLCs, even though the detailed mechanism remains unclear. Since the RIPL peptide is a cell-penetrating and homing peptide, containing an R8 domain [5], non-specific intracellular uptake, including caveolin-mediated endocytosis, is expected. Consequently, we could suggest that the cellular uptake of both RIPL-NLCs and PEG(5)-RIPL-NLCs was energy-dependent and mainly governed by macropinocytosis, although complex mechanisms were involved.

Throughout the in vivo biodistribution study in the plasma and representative organs of mice, treatment with DTX-loaded NLC formulations resulted in higher DTX levels than did that with DTX-Sol, even though the distribution patterns were inconsistent, likely due to DTX encapsulation in NLCs. Owing to the solid state of lipid matrix-type nanocarriers, NLCs undergo slow degradation in the body, which explains the slow, controlled DTX release [40]. However, unexpectedly, the plasma DTX concentration in mice treated with RIPL-NLCs was higher than that in mice administered PEG(5)-RIPL-NLCs. It was considered that the high protein-binding capacity of RIPL-NLCs could affect their blood level. Especially for a highly protein-bound drug, as free drug is cleared by the kidney, the drug–protein complex dissociates, acting as a “depot” that helps maintain more stable plasma levels [41]. In comparison, instead of the total drug measurement, the separated determination of protein-bound, free, and carrier-associated drugs is encouraged [42,43].

The organ distribution results demonstrated a close relationship with the structure of the blood capillary endothelium. DTX accumulation in the lung, which has a continuous endothelial barrier, was remarkably high compared with that in the other organs. DTX-Sol showed high accumulation up to 6 h, while DTX-RIPL-NLCs and DTX-PEG(5)-RIPL-NLCs exhibited decreased accumulation as the time passed, which may be attributed to the fact that the tissue/organ distribution should be accompanied by diffusion of drug molecules across the barrier [44]. Thus, the nanoparticulates DTX-RIPL-NLCs and DTX-PEG(5)-RIPL-NLCs would not support continuous molecular diffusion since the drug molecules should be released from the nanoparticles prior to the diffusional absorption. On the contrary, DTX distribution to the kidney, which has a fenestrated endothelial barrier, was different. DTX-RIPL-NLCs and DTX-PEG(5)-RIPL-NLCs showed higher accumulation than did DTX-Sol, which may be explained by easy excretion of dissolved drug molecules in the urine, whereas the urinary excretion of both NLC formulations should be limited. Meanwhile, compared to DTX-Sol, both NLC formulations exhibited greater accumulation, especially at an earlier time point (0.5 h), in the liver and spleen, which possess a sinusoidal endothelial barrier. However, after 2 h, the accumulation pattern changed, resulting in a relatively high accumulation of DTX-Sol. More importantly, in virtue of PEGylation, DTX-PEG(5)-RIPL-NLCs could avoid the recognition by MPS and showed a reduced uptake by macrophages [45]. Compared with that of DTX-RIPL-NLCs, the accumulation of DTX-PEG(5)-RIPL-NLCs in the liver and spleen was somewhat low and was further reduced after 2 h, particularly in the spleen. This behavior may be considered as proving the stealth effect of PEGylation. However, a pharmacodynamic investigation in a suitable animal cancer model and comprehensive pharmacokinetic studies of both NLC formulations are needed for their practical development in the future.

## 5. Conclusions

Steric stabilization of RIPL-NLCs was successfully achieved by surface modification with PEG3000 at a 5% molar ratio to the total lipids, allowing encapsulation of the hydrophobic drug DTX. Owing to the PEG layer, protein adsorption significantly decreased, and the uptake by macrophages was inhibited, suggesting a possibility of an enhanced stealth effect in vivo. PEG(5)-RIPL-NLCs, with their selectivity for hepsin, showed a superior intracellular uptake by hepsin-expressing cancer cell lines via energy-dependent macropinocytosis. Administration of DTX-loaded NLC formulations resulted in higher plasma DTX levels in mice than did that of DTX-Sol. Meanwhile, the uptake of PEG(5)-RIPL-NLCs was markedly reduced in organs with the reticuloendothelial system. Thus, we conclude that PEG(5)-RIPL-NLCs may be a promising nanocarrier for selective drug targeting with a high payload of poorly water-soluble drugs.

## Figures and Tables

**Table 1:** Physicochemical characteristics of NLCs.

		pNLCs	RIPL-NLCs	PEG-RIPL-NLCs		
				PEG(1)-RIPL-NLCs	PEG(5)-RIPL-NLCs	PEG(10)-RIPL-NLCs
Empty	Size (nm)	132.1 ± 1.2	271.8 ± 8.4	230.3 ± 1.4	212.0 ± 2.7	200.1 ± 5.9
	PDI	0.21 ± 0.08	0.25 ± 0.02	0.38 ± 0.07	0.33 ± 0.02	0.33 ± 0.07
	ZP (mV)	−16.5 ± 0.4	6.8 ± 0.6	1.5 ± 0.2	−3.67 ± 0.1	−5.3 ± 0.2
DiI-loaded	Size (nm)	130.9 ± 2.1	277.4 ± 3.3	238 ± 5.3	207.1 ± 4.2	199.5 ± 6.8
	PDI	0.16 ± 0.05	0.28 ± 0.03	0.32 ± 0.01	0.32 ± 0.01	0.31 ± 0.03
	ZP (mV)	−14.1 ± 0.5	9.9 ± 0.2	3.0 ± 0.6	−4.5 ± 0.5	−6.6 ± 0.4
	EE (%)	99.7 ± 0.1	99.6 ± 0.1	99.5 ± 0.1	99.5 ± 0.1	99.5 ± 0.1
	DL (μg/mg)	1.9 ± 0.1	2.0 ± 0.1	2.1 ± 0.1	2.0 ± 0.1	2.1 ± 0.1
DTX-loaded	Size (nm)	132.5 ± 2.2	260.2 ± 4.5	233.1 ± 3.5	216.4 ± 4.3	195.8 ± 2.7
	PDI	0.24 ± 0.01	0.27 ± 0.01	0.25 ± 0.02	0.24 ± 0.03	0.32 ± 0.01
	ZP (mV)	−17.7 ± 1.2	7.8 ± 0.9	1.9 ± 0.2	−6.3 ± 0.6	−7.8 ± 0.3
	EE (%)	93.9 ± 0.2	94.5 ± 0.4	94.0 ± 1.0	91.8 ± 0.3	94.8 ± 0.5
	DL (μg/mg)	108.0 ± 2.4	109.1 ± 1.9	105.3 ± 2.6	102.3 ± 2.3	104.0 ± 5.9

Data represent the mean ± SD (*n* = 3).
